# Supplementary material for: MC BTS: Simultaneously Resolving Magnetization Transfer Effect and Relaxation for Multiple Components
Source: Magn Reson Med. 2025 Dec 16;95(5):2538–53. doi: 10.1002/mrm.70179 (PMC12962219; doi:10.1002/mrm.70179)
Supplement: Supplementary file 1 — Appendix S1: Comparison of simulated signals with experimentally acquired data. Appendix S2: Noise performance comparison between 3‐pool MC BTS and 2‐pool BTS. Appendix S3: MC BTS sensitivity analysis. Appendix S4: Effect of MT asymmetry on MC BTS signal. Appendix S5: Effect of MT asymmetry on MC BTS quantification. Appendix S6: Effect of Super‐Lorentzian on‐resonance cut‐off frequency on MC BTS quantification. [file MRM-95-2538-s001.docx]

MC BTS: simultaneously resolving magnetization transfer effect and relaxation for multiple – Supplementary Information

S1: Comparison of simulated signals with experimentally acquired data

Simulated signals from representative regions of interest were generated using the sequence parameters and quantification values obtained in both in vivo brain and knee studies (Figure S1). Across all flip angles and echo times, the simulated signals show good agreement with the experimentally measured data in both anatomies.

**Figure S1**: Simulated signals compared with experimentally acquired data from representative regions in both the brain (top row) and knee (bottom row) in vivo studies. Good agreement is observed across all flip angles and echo times.

S2: Noise performance comparison between 3-pool MC BTS and 2-pool BTS

A minimum least squares residuals (MLSR) calculation was carried out by fixing the model parameter of interest and varying all other model parameters, with the minimum least squares residual designated as the MLSR^1^, for both 3-pool MC BTS and 2-pool BTS models. Comparing common parameters slow-relaxing free pool longitudinal relaxation (*T*_1_^S^) and macromolecular proton fraction (*f*_R_), *f*_R_ shows similar cost signature whereas *T*_1_^S^ exhibits higher concavity in the 2-pool model compared to the 3-pool, as shown in the following figure. This reflects the complexity and high number of parameters of the three-pool MC BTS model.

**Figure S2**: Minimum least squares residual calculated for (left) macromolecular proton fraction *f*_R_ and (right) slow-relaxing free water longitudinal relaxation *T*_1_^S^ using 3-pool MC-BTS model (blue) and 2-pool BTS model (red).

S3: MC BTS sensitivity analysis

A sensitivity analysis was conducted to understand which of the many sequence encoding mechanisms contributes most to the quantification of our model parameters. Utilizing the sequence and model parameters employed in our Monte Carlo simulations, derivatives of model parameter with respect to encoding mechanism was calculated. The encoding mechanisms analyzed were excitation flip angle and offset frequency. Our results show that excitation flip angle effects our model parameters quantification the most, which is likely due its influence on all the steady-state signals used for quantification. By comparison, offset frequency influences only the BTS acquisitions (acquisitions with BTS pulse applied), which accounts for ~39% of all steady-state signals used. The results are provided below.

**Table S1.**

|  | *T*_1_^F^ | *T*_1_^S^ | *T*_1_^R^ | *f*_F_ | *f*_S_ | *f*_R_ | *T*_2_^F*^ | *T*_2_^S*^ |
| --- | --- | --- | --- | --- | --- | --- | --- | --- |
| Flip Angle ($\alpha$) | $\frac{d}{d\alpha}$ *T*_1_^F^ = -181.7 | $\frac{d}{d\alpha}$ *T*_1_^S^ = -246.3 | $\frac{d}{d\alpha}$ *T*_1_^R^ = -184.2 | $\frac{d}{d\alpha}$ *f*_F_ = 29.7 | $\frac{d}{d\alpha}$ *f*_S_ = 11.6 | $\frac{d}{d\alpha}$ *f*_R_ = 71.2 | $\frac{d}{d\alpha}$ *T*_2_^F*^ = 1.2 | $\frac{d}{d\alpha}$ *T*_2_^S*^ = 2.8 |
| Offset Frequency ($\Delta$) | $\frac{d}{d\Delta}$ *T*_1_^F^ = 0.127 | $\frac{d}{d\Delta}$ *T*_1_^S^ = 0.052 | $\frac{d}{d\Delta}$ *T*_1_^R^ = 0.037 | $\frac{d}{d\Delta}$ *f*_F_ = -0.019 | $\frac{d}{d\Delta}$ *f*_S_ = -0.011 | $\frac{d}{d\Delta}$ *f*_R_ = -0.000 | $\frac{d}{d\Delta}$ *T*_2_^F*^ = -0.001 | $\frac{d}{d\Delta}$ *T*_2_^S*^ = -0.002 |

S4: Effect of MT asymmetry on MC BTS signal

A simulation study was carried out incorporating a 2.34 ppm upfield shift of the macromolecular pool^2^ to investigate the effect of MT asymmetry on the MC BTS signal. Using the same spin and sequence parameters described in the *Simulations* section. Two sets of simulations, one with ∆_off_ = 4 kHz and another with ∆_off_ = -4 kHz applied, were carried out by sweeping the excitation flip angle from 1 ~ 90º (Figure S3). Within experimentally feasible flip angles (1º ~ 40º) and at frequency offset ∆_off_ = 4 kHz, the maximum deviations between the simulated data and analytical equations of the transverse magnetization were 3.16% and 1.65% for the fast-relaxing and slow-relaxing pools, and 2.45% and 0.69% for BL. When applying ∆_off_ = -4 kHz, maximum deviations were 3.15% and 1.63% for the fast-relaxing and slow-relaxing pools, and 2.45% and 0.69% for BL. In comparison, when MT asymmetry was not incorporated, the deviations were 3.15% and 1.64% for the fast-relaxing and slow-relaxing pools, and 2.45% and 0.69% for BL.

**Figure S3**: Incorporating a 2.34 ppm upfield shift of the macromolecular pool^2^ and sweeping the excitation flip angle from 1º ~ 90º, for both offset frequencies ∆_off_ = ±4 kHz, simulation results show that MT asymmetry has minimum effect on signal, both agreeing well with signal generated without MT asymmetry for both BTS and BL acquisitions.

S5: Effect of MT asymmetry on MC BTS quantification

Monte Carlo simulations (SNR = 50, n = 50,000 observations) incorporating a 2.34 ppm upfield shift of the macromolecular pool^2^ were conducted using the same spin and sequence parameters described in the *Simulations* section, for offset frequencies ∆_off_ = ±4 kHz. As summarized in the table below, quantification results at both offsets are in good agreement with each other and with the ground truth, consistent with the above finding that MT asymmetry has a minimal effect on the signal. For comparison, Monte Carlo results obtained without incorporating MT asymmetry (symmetric MT) are also included.

**Table S2.^1^**

|  | *T*_1_^F^ [s] | *T*_1_^S^ [s] | *T*_1_^R^ [s] | *f*_F_ [%] | *f*_S_ [%] | *f*_R_ [%] | *k*_FR_ [s^-1^] | *k*_SR_ [s^-1^] | *k*_SF_ [s^-1^] | *T*_2_^F*^ [ms] | *T*_2_^S*^ [ms] |
| --- | --- | --- | --- | --- | --- | --- | --- | --- | --- | --- | --- |
| Ground Truth | 0.35 | 0.8 | 0.257 | 21.7 | 65.1 | 13.2 | 1.5 | 3.1 | 3.33 | 10 | 40 |
| Asymmetric MT +4 kHz | 0.345±0.013 | 0.794±0.023 | 0.261±0.016 | 21.5±0.6 | 65.7±0.7 | 12.8±0.3 | 1.456±0.039 | 3.009±0.081 | 3.304±0.099 | 10±0.04 | 40±0.10 |
| Asymmetric MT -4 kHz | 0.348±0.012 | 0.812±0.023 | 0.265±0.015 | 21.2±0.6 | 64.9±0.6 | 13.8±0.3 | 1.659±0.039 | 3.242±0.081 | 3.264±0.095 | 10±0.04 | 40±0.09 |
| Symmetric MT | 0.345±0.011 | 0.801±0.023 | 0.257±0.015 | 21.4±0.6 | 65.0±0.6 | 13.5±0.3 | 1.539±0.039 | 3.181±0.081 | 3.289±0.089 | 10±0.04 | 40±0.09 |

^1^Data acquired are presented as mean ± SD.

S6: Effect of Super-Lorentzian on-resonance cut-off frequency on MC BTS quantification

In MC BTS, on-resonance saturation effect that occurs during excitation is modelled using the Super-Lorentzian lineshape, which adequately characterizes the saturation of molecules in tissues such as white matter, grey matter and cartilage^3,4^. Due to its singularity at on-resonance, we estimated it based on extrapolation from cut-off frequency $\Delta$ = 1 kHz to the asymptotic limit $\Delta$ 🡪 0^5^. To investigate the effect of using of a wider cut-off frequency such as $\Delta$ = 2 kHz, we estimated the on-resonance singularity of the Super-Lorentzian from $\Delta$ = 2 kHz to $\Delta$ 🡪 0 and regenerated the quantification maps, shown in the following table.

**Table S3.^1^**

| ROI |  | cut-off frequency ∆ = 1 kHz^2^ | | | | | | | | | | |  | cut-off frequency ∆ = 2 kHz^3^ | | | | | | | | | | |
| --- | --- | --- | --- | --- | --- | --- | --- | --- | --- | --- | --- | --- | --- | --- | --- | --- | --- | --- | --- | --- | --- | --- | --- | --- |
|  |  | *T*_1_^F^ [s] | *T*_1_^S^ [s] | *T*_1_^R^ [s] | *f*_F_ [%] | *f*_S_ [%] | *f*_R_ [%] | *k*_FR_ [s^-1^] | *k*_SR_ [s^-1^] | *k*_SF_ [s^-1^] | *T*_2_^F*^ [ms] | *T*_2_^S*^ [ms] |  | *T*_1_^F^ [s] | *T*_1_^S^ [s] | *T*_1_^R^ [s] | *f*_F_ [%] | *f*_S_ [%] | *f*_R_ [%] | *k*_FR_ [s^-1^] | *k*_SR_ [s^-1^] | *k*_SF_ [s^-1^] | *T*_2_^F*^ [ms] | *T*_2_^S*^ [ms] |
|  |  |  |  |  |  |  |  |  |  |  |  |  |  |  |  |  |  |  |  |  |  |  |  |  |
| WM region | | | | | | | | | | | | | | | | | | | | | | | | |
|  | | | | | | | | | | | | | | | | | | | | | | | | |
| Corpus   callosum,   genu |  | 0.285 ± 0.010 | 0.96 ± 0.21 | 0.785 ± 0.06 | 13.2 ± 5.1 | 70.8 ± 6.0 | 16.0 ± 3.1 | 2.69 ± 1.10 | 3.69 ± 0.72 | 7.14 ± 3.47 | 12 ± 1 | 57 ± 13 |  | 0.285 ± 0.010 | 0.96 ± 0.21 | 0.786 ± 0.06 | 12.7 ± 5.1 | 71.6 ± 6.0 | 15.8 ± 3.1 | 3.15 ± 0.61 | 3.64 ± 0.71 | 7.59 ± 3.08 | 12 ± 1 | 57 ± 13 |
|  |  |  |  |  |  |  |  |  |  |  |  |  |  |  |  |  |  |  |  |  |  |  |  |  |
| Corpus   callosum,   splenium |  | 0.283 ± 0.006 | 1.341 ± 0.33 | 0.87 ± 0.07 | 9.0 ± 3.5 | 77.6 ± 5.5 | 13.4 ± 2.8 | 2.15 ± 0.92 | 3.08 ± 0.65 | 4.65 ± 2.07 | 11 ± 1 | 51 ± 8 |  | 0.282 ± 0.006 | 1.339 ± 0.34 | 0.86 ± 0.08 | 8.6 ± 3.3 | 78.3 ± 5.2 | 13.2 ± 2.8 | 2.64 ± 0.55 | 3.04 ± 0.64 | 5.14 ± 1.99 | 12 ± 1 | 50 ± 8 |
|  | | | | | | | | | | | | | | | | | | | | | | | | |
|  |  |  |  |  |  |  |  |  |  |  |  |  |  |  |  |  |  |  |  |  |  |  |  |  |
| Frontal white   matter |  | 0.282 ± 0.006 | 1.23 ± 0.39 | 0.83 ± 0.07 | 9.5 ± 3.9 | 78.0 ± 6.5 | 12.5 ± 3.2 | 2.12 ± 0.94 | 2.88 ± 0.73 | 5.12 ± 2.48 | 12 ± 1 | 63 ± 13 |  | 0.281 ± 0.006 | 1.28 ± 0.45 | 0.84 ± 0.08 | 8.9 ± 4.0 | 79.2 ± 7.0 | 11.9 ± 3.5 | 2.38 ± 0.70 | 2.74 ± 0.81 | 5.34 ± 2.42 | 13 ± 1 | 63 ± 13 |
|  | | | | | | | | | | | | | | | | | | | | | | | | |
| GM region | | | | | | | | | | | | | | | | | | | | | | | | |
|  | | | | | | | | | | | | | | | | | | | | | | | | |
| Caudate   nucleus |  | 0.278 ± 0.004 | 1.60 ± 0.36 | 0.86 ± 0.07 | 6.7 ± 2.4 | 85.9 ± 4.2 | 7.5 ± 2.3 | 1.34 ± 0.59 | 1.72 ± 0.53 | 3.69 ± 1.54 | 13 ± 1 | 61 ± 13 |  | 0.278 ± 0.004 | 1.60 ± 0.36 | 0.85 ± 0.06 | 7.0 ± 2.4 | 84.6 ± 4.9 | 8.4 ± 3.1 | 1.67 ± 0.62 | 1.94 ± 0.71 | 4.20 ± 1.46 | 13 ± 1 | 61 ± 13 |
|  | | | | | | | | | | | | | | | | | | | | | | | | |
| Cerebral   cortex |  | 0.274  ± 0.009 | 1.65 ± 0.53 | 0.88 ± 0.09 | 6.5 ± 3.5 | 86.3 ± 6.1 | 7.2 ± 3.6 | 1.19 ± 0.81 | 1.66 ± 0.83 | 3.46 ± 2.24 | 13 ± 1 | 83 ± 24 |  | 0.274  ± 0.009 | 1.66 ± 0.53 | 0.87 ± 0.09 | 6.4 ± 3.7 | 86.2 ± 6.6 | 7.3 ± 3.9 | 1.47 ± 0.78 | 1.68 ± 0.90 | 3.86 ± 2.23 | 13 ± 1 | 82 ± 25 |
|  | | | | | | | | | | | | | | | | | | | | | | | | |
| Thalamus |  | 0.277  ± 0.004 | 1.72 ± 0.48 | 0.91 ± 0.10 | 6.2 ± 2.8 | 85.0 ± 4.4 | 8.8 ± 2.1 | 1.75 ± 0.42 | 2.01 ± 0.49 | 3.75 ± 1.67 | 12 ± 1 | 56 ± 10 |  | 0.277  ± 0.004 | 1.72 ± 0.49 | 0.90 ± 0.11 | 6.2 ± 2.8 | 85.1 ± 4.4 | 8.7 ± 2.1 | 1.74 ± 0.42 | 2.00 ± 0.48 | 3.73 ± 1.67 | 12 ± 1 | 56 ± 10 |

^1^Data acquired are presented as mean ± SD.

^2^Super-Lorentzian on-resonance singularity estimated as extrapolation from 1 kHz to the asymptotic limit ∆ 🡪 0.

^3^Super-Lorentzian on-resonance singularity estimated as extrapolation from 2 kHz to the asymptotic limit ∆ 🡪 0.

1. Bouhrara M, Reiter DA, Celik H, Fishbein KW, Kijowski R, Spencer RG. Analysis of mcDESPOT- and CPMG-derived parameter estimates for two-component nonexchanging systems. *Magn Reson Med*. 2016;75(6):2406-2420. doi:https://doi.org/10.1002/mrm.25801

2. Hua J, Jones CK, Blakeley J, Smith SA, van Zijl PCM, Zhou J. Quantitative description of the asymmetry in magnetization transfer effects around the water resonance in the human brain. *Magn Reson Med*. 2007;58(4):786-793. doi:https://doi.org/10.1002/mrm.21387

3. Morrison C, Mark Henkelman R. A Model for Magnetization Transfer in Tissues. *Magn Reson Med*. 1995;33(4):475-482. doi:10.1002/mrm.1910330404

4. Liu F, Block WF, Kijowski R, Samsonov A. Rapid multicomponent relaxometry in steady state with correction of magnetization transfer effects. *Magn Reson Med*. 2016;75(4):1423-1433. doi:10.1002/mrm.25672

5. Gloor M, Scheffler K, Bieri O. Quantitative magnetization transfer imaging using balanced SSFP. *Magn Reson Med*. 2008;60(3):691-700. doi:10.1002/mrm.21705
